# Dataset on granulopoiesis- and lymphopoiesis-stimulating cytokine levels in insulin secretagogue users with incident breast cancer

**DOI:** 10.1016/j.dib.2017.02.027

**Published:** 2017-02-20

**Authors:** Zachary A.P. Wintrob, Jeffrey P. Hammel, George K. Nimako, Dan P. Gaile, Alan Forrest, Alice C. Ceacareanu

**Affiliations:** a State University of New York at Buffalo, Department of Pharmacy Practice, NYS Center of Excellence in Bioinformatics and Life Sciences, 701 Ellicott Street, Buffalo, NY 14203, United States; b Cleveland Clinic, Dept. of Biostatistics and Epidemiology, 9500 Euclid Ave., Cleveland, OH 44195, United States; c State University of New York at Buffalo, Dept. of Biostatistics, 718 Kimball Tower, Buffalo, NY 14214, United States; d The UNC Eshelman School of Pharmacy, Division of Pharmacotherapy and Experimental Therapeutics, Campus Box 7569, Chapel Hill, NC 27599, United States; e Roswell Park Cancer Institute, Dept. of Pharmacy Services, Elm & Carlton Streets, Buffalo, NY 14263, United States

**Keywords:** Hematopoiesis, Hematopoietic cytokines, Secretagogue, GM-CSF, G-CSF, IL-7, Breast cancer, Diabetes, Cancer outcomes, Cancer prognosis

## Abstract

GM-CSF and G-CSF are widely used for their benefit in reducing chemotherapy-associated neutropenia. However, whether GM- or G-CSF administration could have tumorigenic or pro-metastatic effects or whether insulin resistance could negatively impact such effects is not known. Their ability to stimulate monocyte production at the same time with the highly sought after neutrophils’ production, enables an enhanced potential for activation of tumor-associated macrophages. At the same time, IL-7 remains the main driver of B and T cell differentiation and maturation, a process linked to the development of insulin resistance and response to diabetes pharmacotherapy.

Insulin secretagogues have the potential to interfere with the hematopoiesis process, respectively with the formation of lineages that may lead to a tumorigenic or pro-metastatic phenotype, but this relationship has not been yet investigated. The data presented here shows the relationship between pre-existing use of insulin secretagogues in women diagnosed with breast cancer and type 2 diabetes mellitus, the GM-CSF, G-CSF and IL-7 cytokine profiles at the time of breast cancer diagnosis, and subsequent cancer outcomes. A Pearson correlation analysis evaluating the relationship between investigated cytokines stratified by secretagogue use and controls, and interferon is also provided.

**Specifications table**

**Table:** 

Subject area	Clinical and Translational Research
More specific subject area	Biomarker Research, Cancer Epidemiology
Type of data	Tables
How data was acquired	Tumor registry query was followed by vital status ascertainment, and medical records review
	Luminex®-based quantitation of hematopoietic cytokines (granulocyte colony stimulating factor, granulocyte macrophage colony stimulating factor, and interleukin- 7) from plasma samples was conducted.
	A Luminex®200^TM^ instrument with Xponent 3.1 software was used to acquire all data
Data format	Analyzed
Experimental factors	A total of 3 hematopoietic cytokines were determined from the corresponding plasma samples collected at the time of breast cancer diagnosis
Experimental features	The dataset included 97 adult females with diabetes mellitus and newly diagnosed breast cancer (cases) and 194 matched controls (breast cancer only). Clinical and treatment history were evaluated in relationship with cancer outcomes and hematopoietic cytokines profiles. A cytokine correlation analysis was also performed.
Data source location	United States, Buffalo, NY - 42° 53׳ 50.3592"N; 78° 52׳ 2.658"W
Data accessibility	The data is with this article

**Value of the data**

• Lymphopoiesis and granulopoiesis are processes governed by very distinct hematopoiesis cytokines: IL-7, GM-CSF, and G-CSF.
• G-CSF and GM-CSF levels are elevated in type 2 diabetes and breast cancer and have the potential to increase the density of cancer cells at the metastatic sites, resulting in enhanced pro-metastatic ability [2], [4], [5].
• IL-7׳s aberrant expression is thought to be involved with breast cancer development [5].
• This dataset represents the observed relationship between insulin secretagogue use, cancer outcomes and circulating GM-CSF, G-CSF and IL-7 at breast cancer diagnosis.
• Our observations may assist in future research decisions aimed at clarifying the involvement of insulin production in hematopoiesis.

## 1 Data

Reported data represents the observed association between use of insulin secretagogues preceding breast cancer diagnosis and GM-CSF, G-CSF and IL-7 profiles at the time of cancer diagnosis in women with diabetes mellitus (Table 1). Data in Table 2 includes the observed correlations between the investigated biomarkers stratified by type 2 diabetes mellitus pharmacotherapy and controls. Interferon α2 and γ correlation with each of the studied biomarkers is presented in Table 2.

## 2 Experimental design, materials and methods

The present study, aiming to assess the impact of insulin secretagogue use on hematopoietic cytokine profiles linked with BC outcomes, was the result of two protocols approved by both Roswell Park Cancer Institute (EDR154409 and NHR009010) and the State University of New York at Buffalo (PHP0840409E). Eligible plasma specimens banked at the time of diagnosis, prior to initiation of therapy, in the Roswell Park Cancer Institute Data Bank and Bio-Repository were analyzed to establish hematopoietic cytokine profiles which were subsequently linked with demographic and clinical patient information retrieved from patient records.

### 2.1 Study population

A review of the medical and pharmacotherapy history of all incident of breast cancer diagnoses made at Roswell Park Cancer Institute between 01/01/2003 and 12/31/2009 were considered for inclusion (n=2194). The medical and pharmacotherapy history review established the presence of type II diabetes mellitus at baseline, thereby identifying patients having a diagnosis of diabetes prior to the incident breast cancer diagnosis.

### 2.2 Inclusion and exclusion criteria

In order to be eligible for inclusion the subject must have had a treatment-naïve plasma sample (meaning that it was collected prior to the initiation of any cancer-related therapy - surgery, radiation or pharmacotherapy) available in the Institute׳s Data Bank and Bio-Repository. The subject had to be at least 18 years old and female. Additionally, all subjects included as cases must have had pre-existing diabetes at breast cancer diagnosis. Furthermore, control subjects had to meet all the same criteria with the exception of a pre-existing diabetes diagnosis which would, in fact, rendered them an eligible case.

Exclusion criteria included prior cancer history, unclear date of diagnosis, incomplete clinical records, type 1 or unclear diabetes status, and male sex. An exact list of exclusions and numbers of excluded subjects by criteria is available in the original research article by Wintrob et al. [1]. Out of 2194 incident breast cancer cases identified in the study window, 97 were eligible for inclusion in this analysis.

### 2.3 Control-matching approach

A 2:1 control to case matching ratio was employed. For each of the 97 adult female subjects with breast cancer and diabetes mellitus (defined as “cases”), two other female subjects diagnosed with breast cancer but lacking a pre-existing diabetes mellitus (defined as “controls”) were included. Matching was performed according to the following criteria: age at diagnosis, body mass index category, ethnicity, menopausal status, sample storage time, and tumor stage (as per the American Joint Committee on Cancer). The matching criteria were applied according to the methods outlined in Wintrob et al. [1].

### 2.4 Demographic and clinical data collection

Documentation of the clinical and treatment history was conducted as previously described [1]. The Institute׳s Tumor Registry provided the vital status for each subject. The Institute׳s Tumor Registry maintains a database, which is updated biannually by querying the National Comprehensive Cancer Networks’ Oncology Outcomes Database for all registered patients. The recorded outcomes included breast cancer recurrence and/or death. Subjects were assigned to treatment strata according to the mechanism of action of the prescribed diabetes pharmacotherapy. The “any secretagogue” group consisted of subjects taking any of the following pharmacotherapies alone or in combination with any other diabetes medication: sulfonylureas (glimepiride, glipizide, glyburide), meglitinides (nateglinide, repaglinide), alpha-glucosidase inhibitors (acarbose, miglitol), and glucagon-like peptide-1 receptor agonists (exenatide, liraglutide). Subjects in the “no secretagogue” strata were taking one or more of the following medications, but none the medications listed above as “secretagogues”: biguanides (metformin), thiazolidinediones (pioglitazone, rosiglitazone), and injectable insulin (rapid-, fast-, intermediate- or long-acting insulin) or no oral pharmacotherapy (diet control alone) [1]. Of note is the fact that 11 of the “any secretagogue” users were receiving injectable insulin, while 9 of the “no secretagogue” group also received insulin.

### 2.5 Plasma specimen storage and retrieval

All the plasma specimens retrieved from the Bio-Repository׳s long-term storage were aliquoted into the appropriate vials such that each assay cold be performed from a single vial in order to avoid repeated freeze-thaw cycles. Each subject had an assigned vial color and barcoded. All storage vials, in addition to being color coded, were barcoded. The consideration of time in long-term storage as a matching criteria ensured that the case and matched control specimens had similar overall storage conditions. Between the retrieval of the specimens from the biobank and assay, only two freeze-thaws were allowed, specifically: the aliquoting step and actual assay.

### 2.6 Luminex^®^ assays

Granulocyte colony stimulating factor, granulocyte macrophage colony stimulating factor, and interleukin- 7 were quantified. Assays were performed according to the manufacturer protocol. The assay used was the HCYTOMAG-60K Luminex^®^ biomarker panel (Millipore Corporation, Billerica, MA). Of note, at the time of sample analysis, macrophage colony stimulating factor assays were not marketed.

### 2.7 Biomarker-pharmacotherapy association analysis

Each assayed biomarker underwent cut-point. The continuous independent variable, the set of a measured biomarker׳s concentrations, were subdivided into the two groups that optimized the log rank test and yielded a minimum of 10 patients in any resulting group. Quartiles were also constructed. The biomarker quartiles and optimized two categories were then tested for association with type 2 diabetes mellitus therapy and controls by Fisher׳s exact test. The Kruskall-Wallis test assessed associations between the continuous biomarker levels and diabetes therapy and contro while the Wilcoxon rank sum assessed pairwise comparisons of treatment levels. Additionally multivariate adjustments for age, tumor stage, body mass index, estrogen receptor status, and cumulative comorbidity were performed. The biomarker analysis was performed using R Version 2.15.3. Please see the original article for an illustration of the analysis workflow [1].

Subsequently, Pearson correlations between biomarkers stratified by type 2 diabetes mellitus pharmacotherapy and controls were assessed. Correlation models were constructed both with and without adjustment for age, body mass index, and the combined comorbidity index. Correlation analyses were performed using SAS Version 9.4.

## Funding sources

This research was funded by the following grant awards: Wadsworth Foundation Peter Rowley Breast Cancer Grant awarded to A.C.C. (UB Grant Number 55705, Contract CO26588).

## Figures and Tables

**Table 1:** Hematopoietic cytokine associations with Secretagogue use.

Biomarker	Biomarker Grouping	Concentration	Control	No Secretagogue	Any Secretagogue	Unadjusted p-value (MVP)			
						p^1^	p^2^	p^3^	Global test
G-CSF (pg/ml)	Median (25th–75th)	–	30.82 (20.84–47.23)	42.02 (29.23–62.95)	34.06 (24.59–61.31)	0.009 (0.380)	0.170 (0.360)	0.380 (0.230)	0.024 (0.420)
	Quartiles	1.60 to 21.73	55 (28.4%)	6 (12.8%)	12 (24.0%)	0.023	0.350	0.520	0.090
		21.84 to 33.05	52 (26.8%)	10 (21.3%)	11 (22.0%)				
		33.11 to 54.17	48 (24.7%)	13 (27.7%)	11 (22.0%)				
		54.29 to 2182.70	39 (20.1%)	18 (38.3%)	16 (32.0%)				
	OS-Based Optimization	1.60 to 12.99	22 (11.3%)	3 (6.4%)	3 (6.0%)	0.430 (0.580)	0.280 (0.870)	1.000 (0.980)	0.500 (0.810)
		**13.06 to 2182.70**	172 (88.7%)	44 (93.6%)	47 (94.0%)				
	DFS-Based Optimization	1.60 to 12.99	22 (11.3%)	3 (6.4%)	3 (6.0%)	0.430 (0.580)	0.280 (0.870)	1.000 (0.980)	0.500 (0.810)
		**13.06 to 2182.70**^a^	172 (88.7%)	44 (93.6%)	47 (94.0%)				
									
GM-CSF (pg/ml)	Median (25th–75th)	–	4.95 (3.48–9.08)	5.66 (3.30–12.97)	5.71 (3.77–8.75)	0.320 (0.080)	0.430 (0.650)	0.750 (0.310)	0.500 (0.160)
	Quartiles	0.64 to 3.46	49 (25.3%)	14 (29.8%)	11 (22.0%)	0.300	0.420	0.380	0.360
		3.52 to 5.29	54 (27.8%)	7 (14.9%)	11 (22.0%)				
		5.32 to 9.50	44 (22.7%)	11 (23.4%)	17 (34.0%)				
		9.64 to 1196.39	47 (24.2%)	15 (31.9%)	11 (22.0%)				
	OS-Based Optimization	0.64 to 2.10	20 (10.3%)	3 (6.4%)	3 (6.0%)	0.580 (0.400)	0.430 (0.460)	1.000 (0.880)	0.610 (0.580)
		**2.20 to 1196.39**	174 (89.7%)	44 (93.6%)	47 (94.0%)				
	DFS-Based Optimization	0.64 to 3.00	35 (18.0%)	8 (17.0%)	7 (14.0%)	0.870 (0.840)	0.500 (0.570)	0.680 (0.650)	0.800 (0.840)
		**3.00 to 1196.39**	159 (82.0%)	39 (83.0%)	43 (86.0%)				
									
IL-7 (pg/ml)	Median (25th–75th)	–	0.58 (0.36–1.76)	1.40 (0.50–3.67)	0.74 (0.40–1.76)	0.003 (0.002)	0.220 (0.790)	0.090 (0.016)	0.010 (0.006)
	Quartiles	0.19 to 0.38	52 (26.8%)	11 (23.4%)	13 (26.0%)	0.047	0.500	0.240	0.110
		0.45 to 0.58	59 (30.4%)	8 (17.0%)	11 (22.0%)				
		0.66 to 1.99	41 (21.1%)	9 (19.1%)	15 (30.0%)				
		2.05 to 70	42 (21.6%)	19 (40.4%)	11 (22.0%)				
	OS-Based Optimization	**0.19 to 0.96**	127 (65.5%)	21 (44.7%)	29 (58.0%)	0.010 (0.024)	0.330 (0.790)	0.190 (0.160)	0.029 (0.070)
		0.98 to 70	67 (34.5%)	26 (55.3%)	21 (42.0%)				
	DFS-Based Optimization	**0.19 to 0.96**	127 (65.5%)	21 (44.7%)	29 (58.0%)	0.010 (0.024)	0.330 (0.790)	0.190 (0.160)	0.029 (0.070)
		0.98 to 70	67 (34.5%)	26 (55.3%)	21 (42.0%)				

a Overall survival (OS)- and disease-free survival (DFS)-optimized biomarker ranges associated with poorer outcomes are represented in bold. Unadjusted p-values: p1, compares *no secretagogue versus control*; p2, compares *any secretagogue versus control*; p3, compares *any secretagogue versus no secretagogue* (as per Kruskal-Wallis test); global test, compares *all categories* (as per Wilcoxon, type 3 error test); MVP, denotes the p-value of each multivariate adjusted analysis corresponding to the earlier described unadjusted analyses. For more information, please see Section 2.7 below and our previously published analysis work flow^1^. MVP=p-value of the multivariate adjusted analysis. Granulocyte colony stimulating factor (G-CSF), granulocyte macrophage colony stimulating factor (GM-CSF), and interleukin- 7 (IL-7).

**Table 2:** Hematopoietic cytokine correlations by Secretagogue use.

Compared Biomarkers		Group	Unadjusted correlation			Adjusted correlation		
			Pearson correlation	95% Confidence interval	p-value	Pearson correlation	95% Confidence interval	p-value
G-CSF	GM-CSF	All Subjects (n=291)	**0.850**	**0.814 to 0.879**	**<0.001**	**0.850**	**0.814 to 0.880**	**<0.001**
		Controls (n=194)	**0.945**	**0.928 to 0.958**	**<0.001**	**0.945**	**0.927 to 0.958**	**<0.001**
		No Secretagogue (n=43)	**0.844**	**0.728 to 0.913**	**<0.001**	**0.854**	**0.739 to 0.920**	**<0.001**
		Any Secretagogue (n=54)	−0.026	−0.291 to 0.244	0.854	−0.001	−0.277 to 0.274	0.993
								
G-CSF	IL-7	All Subjects (n=291)	**0.210**	**0.097 to 0.317**	**<0.001**	**0.214**	**0.101 to 0.322**	**<0.001**
		Controls (n=194)	**0.193**	**0.054 to 0.325**	**0.007**	**0.200**	**0.060 to 0.332**	**0.005**
		No Secretagogue (n=43)	**0.729**	**0.549 to 0.844**	**<0.001**	**0.737**	**0.552 to 0.853**	**<0.001**
		Any Secretagogue (n=54)	0.151	−0.122 to 0.402	0.274	0.043	−0.235 to 0.315	0.761
								
G-CSF	IFN-α2	All Subjects (n=291)	**0.860**	**0.826 to 0.887**	**<0.001**	**0.861**	**0.828 to 0.888**	**<0.001**
		Controls (n=194)	**0.908**	**0.879 to 0.930**	**<0.001**	**0.907**	**0.878 to 0.929**	**<0.001**
		No Secretagogue (n=43)	**0.870**	**0.772 to 0.928**	**<0.001**	**0.875**	**0.775 to 0.933**	**<0.001**
		Any Secretagogue (n=54)	0.015	−0.254 to 0.282	0.913	0.059	−0.220 to 0.329	0.678
								
G-CSF	IFN-γ	All Subjects (n=291)	**0.461**	**0.365 to 0.547**	**<0.001**	**0.462**	**0.366 to 0.548**	**<0.001**
		Controls (n=194)	**0.505**	**0.392 to 0.603**	**<0.001**	**0.505**	**0.392 to 0.604**	**<0.001**
		No Secretagogue (n=43)	**0.657**	**0.444 to 0.780**	**<0.001**	**0.705**	**0.504 to 0.833**	**<0.001**
		Any Secretagogue (n=54)	−0.053	−0.316 to 0.218	0.701	0.010	−0.267 to 0.285	0.946
								
GM-CSF	IL-7	All Subjects (n=291)	**0.426**	**0.327 to 0.515**	**<0.001**	**0.429**	**0.330 to 0.519**	**<0.001**
		Controls (n=194)	**0.197**	**0.058 to 0.329**	**0.006**	**0.203**	**0.063 to 0.335**	**0.005**
		No Secretagogue (n=43)	**0.754**	**0.587 to 0.860**	**<0.001**	**0.785**	**0.626 to 0.881**	**<0.001**
		Any Secretagogue (n=54)	−0.042	−0.306 to 0.228	0.762	−0.006	−0.281 to 0.270	0.968
								
GM-CSF	IFN-α2	All Subjects (n=291)	**0.953**	**0.941 to 0.962**	**<0.001**	**0.953**	**0.941 to 0.962**	**<0.001**
		Controls (n=194)	**0.966**	**0.956 to 0.975**	**<0.001**	**0.967**	**0.956 to 0.975**	**<0.001**
		No Secretagogue (n=43)	**0.963**	**0.931 to 0.980**	**<0.001**	**0.965**	**0.934 to 0.981**	**<0.001**
		Any Secretagogue (n=54)	**0.918**	**0.862 to 0.952**	**<0.001**	**0.922**	**0.867 to 0.955**	**<0.001**
								
GM-CSF	IFN-γ	All Subjects (n=291)	**0.611**	**0.534 to 0.678**	**<0.001**	**0.612**	**0.534 to 0.679**	**<0.001**
		Controls (n=194)	**0.542**	**0.434 to 0.634**	**<0.001**	**0.543**	**0.434 to 0.636**	**<0.001**
		No Secretagogue (n=43)	**0.813**	**0.678 to 0.895**	**<0.001**	**0.824**	**0.690 to 0.904**	**<0.001**
		Any Secretagogue (n=54)	**0.800**	**0.677 to 0.879**	**<0.001**	**0.804**	**0.678 to 0.884**	**<0.001**
								
IL-7	IFN-α2	All Subjects (n=291)	**0.371**	**0.267 to 0.466**	**<0.001**	**0.370**	**0.266 to 0.466**	**<0.001**
		Controls (n=194)	**0.207**	**0.068 to 0.338**	**0.004**	**0.212**	**0.072 to 0.344**	**0.003**
		No Secretagogue (n=43)	**0.804**	**0.664 to 0.890**	**<0.001**	**0.819**	**0.682 to 0.901**	**<0.001**
		Any Secretagogue (n=54)	**−0.024**	**−0.290 to 0.245**	**0.863**	**0.020**	**−0.257 to 0.294**	**0.890**
								
IL-7	IFN-γ	All Subjects (n=291)	**0.316**	**0.208 to 0.416**	**<0.001**	**0.321**	**0.213 to 0.421**	**<0.001**
		Controls (n=194)	**0.181**	**0.041 to 0.313**	**0.011**	**0.187**	**0.046 to 0.321**	**0.009**
		No Secretagogue (n=43)	**0.568**	**0.323 to 0.742**	**<0.001**	**0.649**	**0.422 to 0.799**	**<0.001**
		Any Secretagogue (n=54)	0.004	−0.264 to 0.271	0.978	0.080	−0.200 to 0.348	0.575

Significant correlations are displayed in bolded text. The differences that are only significant in either adjusted or unadjusted correlations are further denoted by an outline. Granulocyte colony stimulating factor (G-CSF), granulocyte macrophage colony stimulating factor (GM-CSF), interleukin- 7 (IL-7), interferon α 2 (IFN-α2), and interferon γ (IFN-γ).
